# Experience-based co-design informed development of a toolbox to help optimise primary care support during transition from children’s hospice care: HOPSCOTCH study protocol

**DOI:** 10.1136/bmjopen-2025-108660

**Published:** 2025-11-27

**Authors:** Emma Chapman, Sarah Mitchell, Lorna Katharine Fraser, Jordanne Wozencroft, Ben Morris, George Peat, Tagore Charles, Joanna Elverson, Paul Carder, Katie Tallowin, Josefine Magnusson, Lucy Ziegler

**Affiliations:** 1 Academic Unit of Palliative Care, University of Leeds, Leeds, UK; 2 Cicely Saunders Institute, King’s College London, London, UK; 3 PPI representative, England, UK; 4 Department of Social work, Education, and Community Wellbeing, Northumbria University, Newcastle upon Tyne, UK; 5 Noah's Ark Children’s Hospice, London, England, UK; 6 St Oswald’s Hospice and Newcastle upon Tyne Hospitals, Newcastle upon Tyne, UK; 7 NHS West Yorkshire Integrated Care Board, Wakefield, UK; 8 Together for Short Lives, Bristol, England, UK

**Keywords:** PALLIATIVE CARE, Primary Health Care, Adolescent, Hospice and Palliative Care Nursing, Paediatric palliative care

## Abstract

**Abstract:**

**Introduction:**

The HOPSCOTCH study ‘Helping Optimise Primary Care Support During Transition From Children’s Hospice Care’ aims to develop a toolbox to enable engagement of primary care services in the care of young people with life-limiting conditions (LLC) with a specific focus on the point of transition from children’s hospice services.

**Methods and analysis:**

Individual interviews will be held with young people with LLC, their families and healthcare professionals (HCPs). In alignment with Experience Based Co-Design (EBCD) methodology, extracts of film and audio from young people and family interviews will be combined to professionally produce a ‘catalyst film’ highlighting key points and experiences before, during and after the transition from children’s hospice care. Role-specific workshops will be held with young people with LLC, their families and HCPs working in primary care, children’s hospices and adult hospice services. The catalyst film will be used in feedback workshops to prompt prioritisation of key issues to take forward into toolbox development in a shared young people, family and HCP workshop. A documentary analysis of resources currently used to support transition and communication between care settings will support contextual understanding of the transition process. Young people, parents and professionals have shaped and continue to have influence over the study delivery as advisors alongside a multidisciplinary steering committee.

The study design has been guided by the UK Medical Research Council complex intervention framework. Intervention development draws on the principles of EBCD and is theoretically driven by the Behaviour Change Wheel.

**Ethics and dissemination:**

The study is registered with the UK’s Clinical Study Registry (ISCTRN75964234).

Ethical approval was obtained from Wales 3 ethics board on 2 July 2025 (IRAS ID 334486). This study will include ongoing dissemination and knowledge transfer to key audiences (young people, parents, service providers, commissioners) via publications, national bodies, knowledge exchange events, web-based platforms, social media and clinical/academic forums.

**STRENGTHS AND LIMITATIONS OF THIS STUDY**

- The voices of young people with life-limiting conditions (LLC) and their families are made central to the research design by using experience-based co-design methodology.
- The methodology enables a focus on experience of primary care which is an under-researched area for children and young people transitioning from children’s hospice.
- The research is timely in that the recent publication of the 10 Year Health Plan for England promotes new Neighbourhood health models for children and young people with multiple, complex health needs.
- A limitation of our study is that our sample only includes young people with LLC and their families who have accessed hospice services. There will be another population of young people with LLC who, due to geographical limitations, lack of awareness, funding gaps and cultural or language barriers, have not yet engaged with hospice services.
- Participation in experience-based co-design requires capacity to take part in a conversational interview and workshops, potentially excluding the voices of those with intellectual or communication disability.

## Introduction

 There are more than 86 000 children and young people living with a life-limiting condition (LLC) in England. LLCs are those for which there is no reasonable hope of cure and from which children are expected to die.^1^ The numbers of young people (aged 14–25 years) with a LLC in England have risen by 40% between 2009 and 2018, and the number of young people with a LLC eligible for transition from children’s to adult services rose from 16 107 to 24 773 over the same period.^1 2^ This is a rapidly growing population with increasingly complex healthcare needs, more of whom are surviving into adulthood.^2^ The involvement of General Practitioners (GPs) and the wider primary care team has been shown to reduce emergency secondary care use and facilitate person-centred continuity of care for this population.^3 4^ They therefore have a role in improving the care of children and young people with LLCs and their family members, particularly at the point of transition to adult services. However, this can be challenging because care during childhood is typically led by specialist services with limited involvement of primary care.^3^,^6^

In 2022 an amendment to the Human Rights Bill made access to palliative care an explicit human right.^7^ This amendment has the power to drive transformative change in the delivery of palliative care. In the National Health Service (NHS) in England, Integrated Care Boards now have a legal responsibility to commission palliative care services that meet their population need and to provide comprehensive equitable palliative care to all. Young people with LLC are a population with complex palliative care needs, many of whom are not having these needs adequately met.^3 6 8 9^ This is in part due to a change in how palliative care services are delivered for this population. For over 30 years, UK children’s hospices have aimed to deliver a model of care aligned with the WHO recommendations for paediatric palliative care, with involvement from the time of diagnosis with a LLC and continued until death. Children’s hospice care is long-term and includes short respite breaks and family support and can extend from infancy well into adulthood. Young people were not discharged from children’s palliative care services irrespective of age. This model has become unsustainable due to the rapidly growing population of young people with LLCs and their increased life expectancy,^1 2^ necessitating a process of transition for young people from children’s palliative care delivered by hospices to adult services as they approach young adulthood—a process families and young people have described as ‘falling off a cliff’.

Adult palliative care services are configured differently to children’s services and are often orientated to the last days and weeks of life. The median duration between referral to an adult hospice and death is 48 days.^10^ Young people with LLCs do not necessarily fit the referral criteria for adult palliative care or hospice services. Adult palliative care services do not necessarily have the same expertise in management of conditions affecting young people or provide the continuity of care and respite for families which is so critical to coping and sustaining a reasonable quality of life. Furthermore, children with complex LLCs often receive care from a range of specialist paediatric teams, all of which will have a transition process independent from the transition from children’s hospices to adult palliative care. Access to appropriate palliative care for young people with LLC has become precarious and highly inequitable,^5^ compounding other inequalities experienced by this population. Prevalence of LLCs is highest among young people of Pakistani origin and in areas of higher deprivation, with the highest prevalence of LLCs in young people being in the North East of England.^1^

There is growing evidence that primary care involvement is critical to the provision of quality palliative care for this population and that the GP has a key role. However, GPs are infrequently engaged in the care of the young person with a LLC or in any aspect of transition planning from children’s to adult services.^5^ The 2023 National Confidential Enquiry into Patient Outcome and Death (NCEPOD) study ‘The Inbetweeners’ found that 100% of young people were registered with a GP at the time of transition and 79.9% had been previously encouraged to access their GP for healthcare. However, only 4 out of 69 parents said their young person‘s GP was involved in any aspect of the transition process.^11^ There is an urgent need for more proactive, personalised care for those with complex healthcare needs. It is widely recognised that there is variation in the level of engagement with palliative care in primary care, with barriers identified including inconsistent skills and training, limited workforce, time and resources.^11 12^ A shift of care from ‘hospital to community’ is a key theme of the recent 10-year health plan for England.^13^ A core function of Neighbourhood Health is proactive identification of patients who could benefit from personalised care and continuity, both key characteristics of good palliative care. A programme of 10 projects on transition undertaken by the UK children’s palliative care charity, Together for Short Lives, found the engagement of GPs is vital to the success of young people transitioning from children’s palliative care services. This work informed the 2023 Transitions Pathway Quality Standards which states ‘Every young person must be supported in adult services by a multi-agency team fully engaged in their care.’^14^

GPs are expert generalists who can hold overall clinical responsibility for their patients, managing uncertainty and clinical risk through continuity. Members of the wider primary care team can be key healthcare professionals for families. Regular GP involvement and continuity of care is associated with reduced emergency secondary care use and better coordination of services.^6^ GPs have the potential to provide many vital aspects of palliative care, including the coordination of care from community and specialist teams, provision of prescriptions and holistic support for family members (including during bereavement). Consistently poor experiences of general practice cause children and families to feel isolated. Young people and families report that fostering a relationship with their general practice enables them to access important aspects of care, including the assessment and management of acute illness, chronic disease, medication reviews and holistic support.^3^,^6^ The National Institute for Health and Care Excellence quality standard for transition from children to adult services,^14^ Together for Short Lives (TFSL) Report, Care Quality Commission (CQC) report ‘From the Pond into the Sea’^15^ and The 2023 NCEPOD study^11^ all state GPs need to be included at an earlier stage in the care of young people with LLC and need to develop their skills particularly in relation to communication with young people with LLCs and their families. GPs have expressed concerns about having an active role in palliative care for young people, including a lack of time, lack of specialist knowledge particularly with rare conditions and understanding of their role alongside specialist colleagues. Barriers can be classified according to three levels: personal: barriers related to knowledge, skills, emotions; relational: barriers concerning communication and collaboration; organisational: barriers related to the organisation of care and compartmentalisation in healthcare.^2 3 5 6^ The HOPSCOTCH study will identify solutions to help address these barriers and facilitate better engagement of General Practice in the care of young adults with LLCs.

## Aim

The study aims are as follows:

1. Understand and identify opportunities to enhance engagement of general practice in the coordination and delivery of care to young people as they transition from children’s hospices to adult services.
2. Co-design a complex intervention (the HOPSCOTCH toolbox) to facilitate this enhanced engagement. Use of the toolbox in clinical practice will be examined in a subsequent feasibility and acceptability trial.

## Objectives

1. To identify opportunities to enhance engagement between children’s hospices and primary care.
2. Identify opportunities for primary care staff to engage with young people with LLC and their family that is feasible and fits within service constraints.
3. We will develop an intervention ‘toolbox’ to support effective engagement using a method adapted from the principles of Experience-Based Co-Design with young people with LLC and their families alongside primary care staff and hospice care staff.

## Methods and analysis

### Patient and public involvement

The origin of this study, its aims, objectives and methodology were born from directly engaging with young people with LLCs, their parents and health professionals who care for this population. Drawing on the views and expertise of young people with LLCs who have experienced transition to adult services has been critical to the development of the protocol, including informing decisions about recruitment and feasibility of the study design. Parents continue to play a key role in coordinating their child’s transition to adult services. Relatedly, previous studies have highlighted the consistent barriers parents face in attempting to coordinate transition. Consequently, it is vital that parents play a role in advising the study. We will form an advisory group of young people with LLCs and their family carers. Recruitment to the advisory group will be supported through links with established groups and organisations such as the ‘Leeds Deep End Research Network’ that represents the views of people from socioeconomically deprived areas in Yorkshire and user-led organisations. To date, we have sought to include the voices and perspectives of young adults and parents of children with LLCs to inform the design and delivery of the study. This has included running a workshop with the user-led organisation Pathfinders Neuromuscular Alliance and a Family Advisory Group to receive crucial feedback pertaining to drafted recruitment and data collection materials (eg, topic guides). This engagement has complemented the roles of the study co-applicants, BM and JW (who have lived experience of transition from children’s hospices) who have been involved in the study design and have key roles in its delivery. Moving forward, our aim is to continue to consult with young adults and parents throughout the project to receive feedback and guidance on, for example, data analysis. We aim to evaluate our Patient and Public Involvement and Engagement strategy using the Public Involvement in Research Impact Toolkit Tracking Tool.^16^ Members will be reimbursed for their involvement in line with National Institute for Health and Care Research (NIHR) guidance.

### Theoretical framework

The study design has been guided by the UK Medical Research Council complex intervention framework^17^ and is theoretically driven by the Behaviour Change Wheel .^18^ Our methodology draws on the principles of Experience-Based Co-Design (EBCD).^19^,^21^ EBCD is a flexible form of qualitative participatory action research and is guided by a framework of six stages; Project set-up, Gathering clinician experiences, Gathering patient and family experience, Joint co-design event, Ongoing resource development work plus Celebration event and review of output.^22^ No power calculation is used to determine sample size in EBCD. Typically, 10–15 participants from each stakeholder group take part. Sample composition is determined by the need to ensure appropriate representation of diverse patients, families and healthcare professionals.

### Consent

We will obtain written and/or verbal consent and agreement from every individual taking part in an interview. Informed consent will be obtained at the start of the interview. For young people under the age of 16, consent will be obtained from their parent and then verbal or written agreement obtained from the young person. In keeping with the Mental Capacity Act, there is an assumption of capacity in young people aged 16 years and over, so they will be asked for consent first, followed by agreement from their parent. This parental agreement is not legally required, but conducting an interview with a young person about a potentially difficult subject without the knowledge of their parents raises potential ethical concerns. For young people aged 18 and above, parental agreement will not be sought. Parental consent will be required for any interviews that require a researcher to visit the family home. Parents and carers who take part as research participants themselves either with or without their young person will be asked for verbal or written consent depending on the mode of interview. Healthcare professionals will give written or verbal consent. Verbal consent involves the researcher reading all questions listed on the consent form and audio recording this and the participants’ yes/no response. Potential participants will be given at least 48 hours to read the participant information sheet. Verbal remote consent will be audio/video recorded and stored separately from the rest of the recorded interview. Participants will be informed of their right to withdraw consent at any time during the interview and to withdraw their data for up to 2 weeks after. Any participant withdrawing from the study will have their data and any personal information destroyed. Consent/assent is incremental so that a person can agree to audio recording of the interview only, audio and video recording (video to be used in intervention co-development process only) or audio and video recording (video will be publicly disseminated in an identifiable way).

### Young person and family perspectives on the transition process and the role of primary care within it

#### Sampling

Young people and family interviews (n=18): six young people and/or family members who have transitioned to adult services, six that are currently transitioning and six that are approaching transition will be invited to participate. We will recruit participants via our children’s hospice partners and the corresponding adult hospices (if the young person has recently transitioned to adult services). We will work with the transition co-ordinators/transition lead at each children’s hospice to identify potential participants who meet the eligibility criteria. To recruit young people and their parent/carer who have already transitioned to adult services, we will liaise via the children’s hospices to share the study information with the transition coordinators at the respective adult hospice.

#### Inclusion criteria (Young people)

- Young person with a LLC.
- Minimum 14 years of age.
- Previous or current user of children’s hospice services.
- The young person must be approaching transition to adult services, in the process of transitioning to adult services or have transitioned to adult services within the previous 5 years.
- Capacity (with support if required) to understand young person information and consent either in written or verbal form.
- Capacity and willingness to take part in a discussion about their experience.

#### Exclusion criteria

- Transition to adult services occurred >5 years ago.
- Unable to provide informed consent (family members can participate instead of the young person if they are unable to provide informed consent).
- Under 14 years of age.
- Young people who are too unwell (as judged by healthcare professionals making initial contact) will not be approached for interview, but their family members may still participate if they wish to.
- Young people who are unable to participate in a conversational interview for any reason related to their condition will not be approached for interview, but their family members may participate if they wish to.

#### Inclusion criteria (Parent or carer)

- Parent/carer from the same household of a young person with a LLC who is approaching transition to adult services, in the process of transitioning to adult services or has transitioned to adult services within the previous 5 years.
- Minimum 18 years of age.
- Parent or carer of a young person who is a previous or current user of children’s hospice services.
- Family/carers may still participate in the study if their young person has died within the previous 5 years and experienced any of the above stages of transition prior to death.
- Capacity to understand information and consent either in written or verbal form.
- Capacity and willingness to take part in a discussion about their experience.

#### Exclusion criteria

- Transition to adult services occurred >5 years ago.
- Unable to provide informed consent.

### Data Collection—Young people and families

In-depth interviews will be conducted by an experienced researcher under the supervision of programme leads. Participants will choose the venue for their interview, where privacy can be assured. Interviews may be carried out by Microsoft Teams or in person, as is the participants’ preference. Participants will be assigned study identification numbers. Demographic forms will be labelled with the participant identification number only. The code link document will be stored on a secure Teams drive at the University of Leeds in its own password protected document.

The interview schedule will be piloted with at least two members of our young person and family advisory board. A blended narrative approach of open narrative followed by semi-structured questions will be used. This approach has the benefit of allowing participants to tell their story regarding their experience of attending children’s hospice and the transition process without imposing a structure or order in the first part of the interview but ensuring that key topics are consistently covered with all participants.

The second substantive section of the interview will use in-depth, semi-structured questions to explore the pathway to transition, the transition process and, if relevant, care beyond transition with a specific focus on the role of the GP practice within it. Interviews will last a maximum of 60 min, to allow sufficient exploration of experience while minimising participant burden. However, because of the narrative approach, it is expected that some interviews may take longer. Interviews will be audio and video recorded if permission is given. Recordings will be transcribed.

### Development of catalyst film

Participants will have consented to the public dissemination and use of their words in a pseudo-anonymised format. An animation will be professionally produced with extracts of pseudo-anonymised interview transcripts. Extracts from audio or video recorded interviews with young people and families’ will also be combined to make a ‘catalyst film’ highlighting overarching themes, key experiences and learning for improvement. Film and animation will highlight key aspects or ‘touch points’ of the transition process. Touch points are emotionally significant moments in the experience. Participants will be given the opportunity to decide if any parts of the interview may not be included and will approve the completed version before dissemination. Our consent process enables participants to choose whether they wish film and audio of them to be used only in workshops during the intervention co-development with other young people, family and healthcare professional participants or also be used in a publicly disseminated film to be widely shared. We will continue to work with young people, familes and patient and public involvement (PPI) partners in an iterative process to determine the content and dissemination strategy for these.

### Feedback workshop 1—Young people and families

Following the analysis of the young people and family interviews, we will hold a feedback workshop for young people and families. Young people and families who took part in the interviews will be invited to the feedback workshop. The purpose of which will be to show the catalyst film and allow the participants to discuss their responses and reactions. The feedback workshop will be facilitated by members of the research team plus at least one PPI co-applicant. The facilitators will run an emotional mapping exercise to help young people identify key points of the transition journey that could benefit from improvement. The meeting will be attended by a professional illustrator who will, in ‘real time’, create storyboards to inform the mapping exercise. From the areas of potential improvement identified by the map, the participants narrow down their list to 4–5 priority issues to be discussed at a joint healthcare and professional co-design event.

### Healthcare professionals’ perspectives on the transition process and their role within it and documentary review

#### Interviews with healthcare professionals (n=18)

Six primary care staff, six adult palliative care clinicians and six paediatric palliative care clinicians will be recruited to participate in interviews which will focus on the pathway to transition, the transition process, documentation supporting this and care beyond transition with a specific focus on the role of primary care within it.

#### Inclusion criteria (healthcare professionals)

- Minimum 18 years of age.
- Work in primary care, adult hospice or children’s hospice.
- Hospice staff have a role or responsibility in supporting transition.
- Capacity to understand information and consent either in written or verbal form.
- Capacity and willingness to take part in a discussion about their experience.

There are no exclusion criteria for healthcare professionals.

Primary care staff will be recruited from practices across West Yorkshire, London and the Northeast of England. Access to West Yorkshire-based practices will be facilitated by the research manager for NHS West Yorkshire Integrated Care Board (PC). Our primary care advisory board members based in the Northeast and Greater London, respectively, will support recruitment of primary care staff in these areas. Our advisory board will make an initial approach and identify primary care staff who are interested in participating in the study. These will be contacted by the researchers who will share any further information they require and, if appropriate, obtain informed consent to take part in an interview. Research activity will be conducted outside of their NHS working time and by University of Leeds Microsoft Teams or on non-NHS property.

Six adult palliative care clinicians and six paediatric palliative care clinicians with a key role or responsibility relating to transition will be recruited via our adult and children’s hospice partners. The children’s hospices are geographically dispersed throughout England and adopt a range of different approaches to transition. Where the hospice has a designated transition lead clinician, their participation in the interview will be requested specifically. As with the primary care staff recruitment process, if palliative care clinicians are interested in participating, the research team will share further information about the study, obtain consent and schedule the interview either at their place of work or online depending on their preference. Interviews lasting approximately 1 hour may be carried out by Microsoft Teams or in person, as is the participants’ preference. Participants will be assigned study identification numbers.

### Data collection

Primary care staff interviews will focus on the transition process and explore potential opportunities to engage with young people with LLC and their families at the time of transition, including those that align to existing primary care services such as medication reviews and learning disability checks. Confidence and perceived competence to provide palliative care to young people with LLCs, and training needs, will be explored. To maximise the feasibility of the intervention, we will explore perspectives about the potential of the primary care multi-disciplinary team who could have a role in transition and how best to engage with them. Interviews with transition leads and palliative care clinicians will focus on the current transition process within their organisations, including barriers and enablers to successful transition and opportunities to engage primary care in the transition process.

### Combined analysis

Interview data will be analysed using reflexive thematic analysis, to draw out key themes that ‘capture something important about the data in relation to the research question and represent some level of patterned response or meaning within the data set’.^23^ Where relevant, young people’s family/carer and professionals’ data will be analysed together; however, data from both groups will also be compared during the development of themes to identify similarities, differences and disagreements. The analysis will focus on barriers and facilitators to providing quality and equitable care at transition, and how different experiences of transition impact on care and quality of life including opportunities for social interaction, psychological support and family support. Other key themes that offer additional insights and contexts will also be included.

The researchers conducting the fieldwork will be the primary analysts, working closely with the project leads and young people. Co-investigators will be involved in the analysis of data to provide a different interpretation from their experience and aid the reflexive process. They will receive appropriate training and support for the role.

Having four analysts working together will help to ensure rigour and dependability of findings.^23^ Analysts 1 and 2 will keep a reflective journal throughout data collection and analysis to record thoughts and reflections on the process and how it may be shaping the responses of participants and interpretation of data. The wider research team and young person and family advisory group will be utilised at key points during the analysis to shape key themes and interpret meaning, to ensure credibility and authenticity of study findings. The five steps of thematic analysis will be applied as follows:

1. Familiarising with the data: analysts 1 and 2 will read and re-read all the transcripts to explore similarities and differences. During this process, the researcher will annotate interesting concepts and ideas (referred to as ‘codes’ from herein) informed by the research objectives and theoretical framework. Analysts 3 and 4 will read a proportion of transcripts, selected to represent some of the diversity in experience and note down commonly occurring codes.
  Working together, the analysts will discuss the selection, labelling and meaning of codes to inform step 2 and decide whether to generate separate codebooks for young person and professional data.
2. Generating initial codes: analysts 1 and 2 will continue to generate codes that represent the data and discuss these regularly with analysts 3 and 4 before applying the agreed codes systematically across the dataset, the purpose of which is to organise the data into meaningful analytical categories. The data will be managed and coded in NVivo software.
3. Searching for themes: using the coded data, the analysts will work together to identify themes by combining groups of codes.
  Analytical tools such as mind mapping and brainstorming will be used during this step, with constant reference to the raw coded data to ensure the meaning is retained. Similarities, differences and disagreements between the young people/family and professionals’ data will also be explored during this step, and a thematic map will be produced to illustrate the links between themes and codes.
  4–5) Reviewing and defining themes: during these steps the analysts will work with the wider research team and young person and family advisory group to review and refine the themes and the thematic map. The final themes will be defined and described using quotations to illustrate meaning and relationships between themes.

### Feedback workshop 2—Healthcare professionals

Following the interview analyses, we will hold a feedback workshop for healthcare professionals. Healthcare professionals who took part in the interviews will be invited to the feedback workshop. The purpose of which will be to feedback to attendees the content of the interviews, review the young person catalyst film/animation and allow the participants to discuss their responses and reactions. The feedback workshop will be facilitated by the research leads, with at least one PPI co-applicant. The facilitators will help healthcare professionals identify the priority areas of potential improvement identified by the data presented. The participants will narrow down their list to 4–5 priority issues to be discussed at a joint healthcare and young person co-design event.

### Documentary review

We will review documentation and map processes currently used by children’s hospices and primary care in relation to the transition process. The documentary review will also incorporate information materials and resources and mechanisms used to share information across organisations. We will adopt the READ approach to documentary review,^24^ a systematic procedure for collecting documents and gaining information from them in the context of health studies at any level (global, national, local).

Sampling: The research team will contact hospices who will share blank copies of documentation used within and across organisations and professional groups in relation to the transition process, via the transition co-ordinator and other relevant professionals. Additional public-facing information such as that accessible through hospice websites will also be collected.

The documentation will be required to meet the following criteria:

#### Documentation inclusion criteria

- Guidelines and policies.
- Relates to the planning of the transition process.
- Staff training materials relating to transition.
- Information materials for staff and young people and their families in relation to transition.
- Flow charts, description of transition pathways.
- Materials used to support communication within and across healthcare organisations in relation to transition.

#### Exclusion criteria

- Documents that have been populated with any identifiable young person or family information.

A digital copy will be saved of every document that meets the inclusion criteria. Data extraction will be undertaken by the researchers and will include the following fields: Author/organisation, date of publication, document type (eg, report, guideline, referral template, planning tool, intended user).

### Analysis of documents

Documentation analysis will involve mapping relevant data onto the nine intervention functions (education, persuasion, incentivisation, coercion, training, restriction, environmental restructuring, modelling and enablement) of the Capability, Opportunity, Motivation, Behaviour (COM-B) model of behaviour change.^20^ This will enable the research team to determine which types of documentation can help support the behaviour change to facilitate an optimal model of transition care and understand where new resources are needed.

### Co-design phase: Joint Experience-Based Co-Design Workshops

A joint young person, carer and healthcare professional 1 day event will be held to co-design the HOPSCOTCH intervention. All young people, families and HCPs that took part in interviews and/or feedback workshops will be invited. This will be held online using University of Leeds Microsoft Teams to facilitate the participation of young people with complex healthcare needs, their family members and HCPs from different regions to come together. It is intended to move participants from feedback to action. Online workshops may be recorded and stored on Microsoft Teams to facilitate note taking.

Methods will draw on the principles of EBCD. The event will be facilitated by stakeholder group leads and an EBCD expert from the Picker Institute, Europe. The event will be structured so that it focuses on the experiences of young people and families, and how they can be improved. The themes and findings and film/animation will be presented. An interactive process mapping exercise will then be undertaken which will be solution-focussed, so that participants can discuss shared priorities and feasible ways that these can be addressed at each stage of the transition process. The discussion and process mapping will lead to the development of a logic model.

Next, structured small group discussions will be held using the logic model to identify and agree key priorities for the HOPSCOTCH toolbox intervention. There will also be an opportunity for participants to contribute any examples of existing good practice and to consider whether these would be further enhanced with the HOPSCOTCH toolbox. Co-design groups will be facilitated by the stakeholder group leads and will include young people, families and clinicians working collectively on creating the HOPSCOTCH intervention with specific focus on the component most relevant to their role in the transition process. The event will close with a presentation by the research team to include a clear plan for outputs, followed by a thank you to participants and a celebration of the day.

The first key output from the co-design event is a logic model to outline optimal primary care support at the time of transition from children’s hospices to adult care, including the roles and responsibilities of young people, family members and professionals from primary care and specialist palliative care. Subsequent co-design online working groups will be facilitated by the stakeholder group leads and will include young people, families and clinicians working collectively on creating aspects of the HOPSCOTCH intervention with specific focus on the component most relevant to their role in the transition process.

This will underpin the development of three ‘modules’ which will make up the HOPSCOTCH toolbox of resources, for further development in a subsequent feasibility and acceptability study:

- **Young person and family module**—information for young people and families regarding the role of the GP at transition and how to engage with them.
- **Primary care module**—a consultation template with embedded links to training and communication resources within existing GP e-learning systems (eg, Royal College of General Practitioners e-learning palliative care) and guidance regarding opportunities for engagement with young people that are feasible and fit within service constraints.
- **Children’s hospice module**—a corresponding template that aligns with the primary care consultation template to improve communication processes and facilitate the flow of information to primary care from the children’s hospice.

## Ethics and dissemination

In contrast to the use of EBCD methodology in individual service development, where ethical approval is often not required, we decided to seek Health Research Authority (HRA) ethical approval for this study. Working with vulnerable young people with LLC and their families does raise ethical concerns of confidentiality, data protection, capacity and potential for causing further distress. EBCD methodology involves the production of identifiable ‘catalyst films’ of participants and it will be essential that young people and their families, who wish to be filmed, fully understand and give recorded consent to the future use and dissemination of these.

Obtaining formal ethical approval allows us to be transparent about how these valid concerns are addressed, satisfy hospice research site governance and overall ensure that the project is carried out ethically and in accordance with principles of good research practice.

Ethical approval was obtained from Wales 3 ethics board 01/07/2025 (IRAS ID 334486).

This study includes ongoing dissemination to key audiences (young people, parents, service providers, commissioners) via knowledge exchange events, web-based platforms, social media and clinical/academic forums.

## Data Availability

No data are available.
