# Supplementary material for: Experience-based co-design informed development of a toolbox to help optimise primary care support during transition from children’s hospice care: HOPSCOTCH study protocol
Source: BMJ Open. 2025 Nov 27;15(11):e108660. doi: 10.1136/bmjopen-2025-108660 (PMC12666166; doi:10.1136/bmjopen-2025-108660)
Supplement: Supplementary file 1 [file bmjopen-2025-108660.reviewer_comments.pdf]

PEER REVIEW HISTORY

BMJ Open publishes all reviews undertaken for accepted manuscripts. Reviewers are asked to complete a checklist review form and are provided with free text boxes to elaborate on their assessment. These free text comments are reproduced below.

ARTICLE DETAILS

Title (Provisional)

HOPSCOTCH Study Protocol: Experience-based co-design informed development of a toolbox to help optimise primary care support during transition from children's care.

Authors

Chapman, Emma; Mitchell, Sarah; Fraser, Lorna Katharine; Wozencroft, Jordanne; Morris, Ben; Peat, George; Charles, Tagore; Elverson, Joanna; Carder, Paul; Tallowin, Katie; Magnusson, Josefine; Ziegler, Lucy

---

This article was not externally reviewed at BMJ Open. Protocols that have been independently assessed prior to submission to BMJ Open are usually fast-tracked to publication on the grounds that further substantial changes will not be possible. This independent assessment will usually be external, independent review for both a substantial grant award from a non-commercial or government funder and ethics approval.
